# Spaced Education and the Importance of Raising Awareness of the Personal Data Protection Act: A Medical Student Population-Based Study

**DOI:** 10.2196/mededu.5586

**Published:** 2016-08-09

**Authors:** Zubin J Daruwalla, Jing L Loh, Chaoyan Dong

**Affiliations:** ^1^ National University Hospital, Singapore Department of Orthopaedic Surgery Singapore Singapore; ^2^ School of Medicine National University, Singapore Singapore Singapore; ^3^ Centre for Medical Education National University, Singapore Singapore Singapore

**Keywords:** medical education, MyDoc, Personal Data Protection Act, secure messaging, spaced education, telehealth, telemedicine

## Abstract

**Background:**

The Personal Data Protection Act (PDPA) of Singapore was first passed in 2012, with subsequent enforcement regulations effective in 2014. Although medical education via digital platforms is not often used in medical schools in Singapore as of yet, many current means of communication at all levels in the medical community from medical schools to clinics to hospitals are unsecure and noncompliant with the PDPA.

**Objective:**

This pilot study will assess the effectiveness of MyDoc, a secure, mobile telehealth application and messaging platform, as an educational tool, secure communications tool, and a tool to raise awareness of the PDPA.

**Methods:**

By replacing current methods of communication with MyDoc and using weekly clinical case discussions in the form of unidentifiable clinical photos and questions and answers, we raised awareness the PDPA among medical students and gained feedback and determined user satisfaction with this innovative system via questionnaires handed to 240 medical students who experienced using MyDoc over a 6-week period.

**Results:**

All 240 questionnaires were answered with very positive and promising results, including all 100 students who were not familiar with the PDPA prior to the study attributing their awareness of it to MyDoc.

**Conclusions:**

Potential uses of MyDoc in a medical school setting include PDPA-compliant student-to-student and student-to-doctor communication and clinical group case discussions with the sharing of patient-sensitive data, including clinical images and/or videos of hospital patients that students may benefit from viewing from an educational perspective. With our pilot study having excellent results in terms of acceptance and satisfaction from medical students and raising awareness of the PDPA, the integration of a secure, mobile digital health application and messaging platform is something all medical schools should consider, because our students of today are our doctors of tomorrow.

## Introduction

Medical education via digital platforms is not often used in medical schools in Singapore as of yet. Rather, traditional rote or “binge and purge” learning often dominates. Examples of more recent and nontraditional methods of teaching include problem-based learning and the flipped classroom model, both of which our local medical school has attempted to adopt. With spaced education having been shown to increase knowledge by up to 50% and strengthen retention by up to two years [1], its potential impact in education and more specifically medical education is significant. This impact could be maximized through increased use of technology in medical schools, providing greater opportunities and avenues from which medical students may benefit, a point reiterated by two studies published just two years ago [2,3]. In the first, it was strongly suggested that medical students learn from real patients by participating in patient care within an educational practice and that their learning is affected by clinicians’ willingness to engage in supportive dialogue; this approach should take place alongside and perhaps ahead of the currently dominant discourse of clinical teaching [2]. The second emphasized the advantages of a sociomaterial approach to practice and learning, stating its specific importance with regard to insights for medical education [3].

The Personal Data Protection Act (PDPA) of Singapore was first passed on October 15, 2012, with subsequent enforcement regulations effective only two years later on July 2, 2014. However, many current means of communication such as social media tools, text messages and hospital messaging system texts are unsecure and do not comply with the PDPA. While at first there may not seem to be any correlation between medical education via a digital health platform and the PDPA, their relationship becomes apparent with the fact that the art and study of medicine inevitably require the sharing of patient information. Although ensuring the use of only nonidentifiable data eliminates the issue of the need for data protection, in medicine this is often not feasible as doctors and students need to be able to identify their patients for various reasons. The primary objective of this study was to deliver medical education in orthopedics through clinical case discussions via the personal digitial platform, MyDoc, and assess the effectiveness of this secure communication and PDPA-compliant platform as an educational tool. The secondary objective was to raise awareness of the PDPA to medical students by determining their reactions to this telehealth platform. We predict that using MyDoc to educate medical students through the use of clinical case discussions will result in improved learning outcomes and raise student awareness of secure messaging and the PDPA in a clinical setting.

## Methods

This was a prospective study that included third-year medical students at the National University of Singapore who agreed to participate. Institutional review board approval was sought; the study was deemed exempt from full review. All students were able to understand, speak, and read English. An oral presentation on MyDoc with mention of the PDPA was given to all students with a subsequent email sent to all participants and a second one to all class representatives. Of 300 third-year medical students, 240 (80.0%) participated in the study, including 116 males and 124 females, all between the ages of 20 and 23 years.

**Figure 1:**
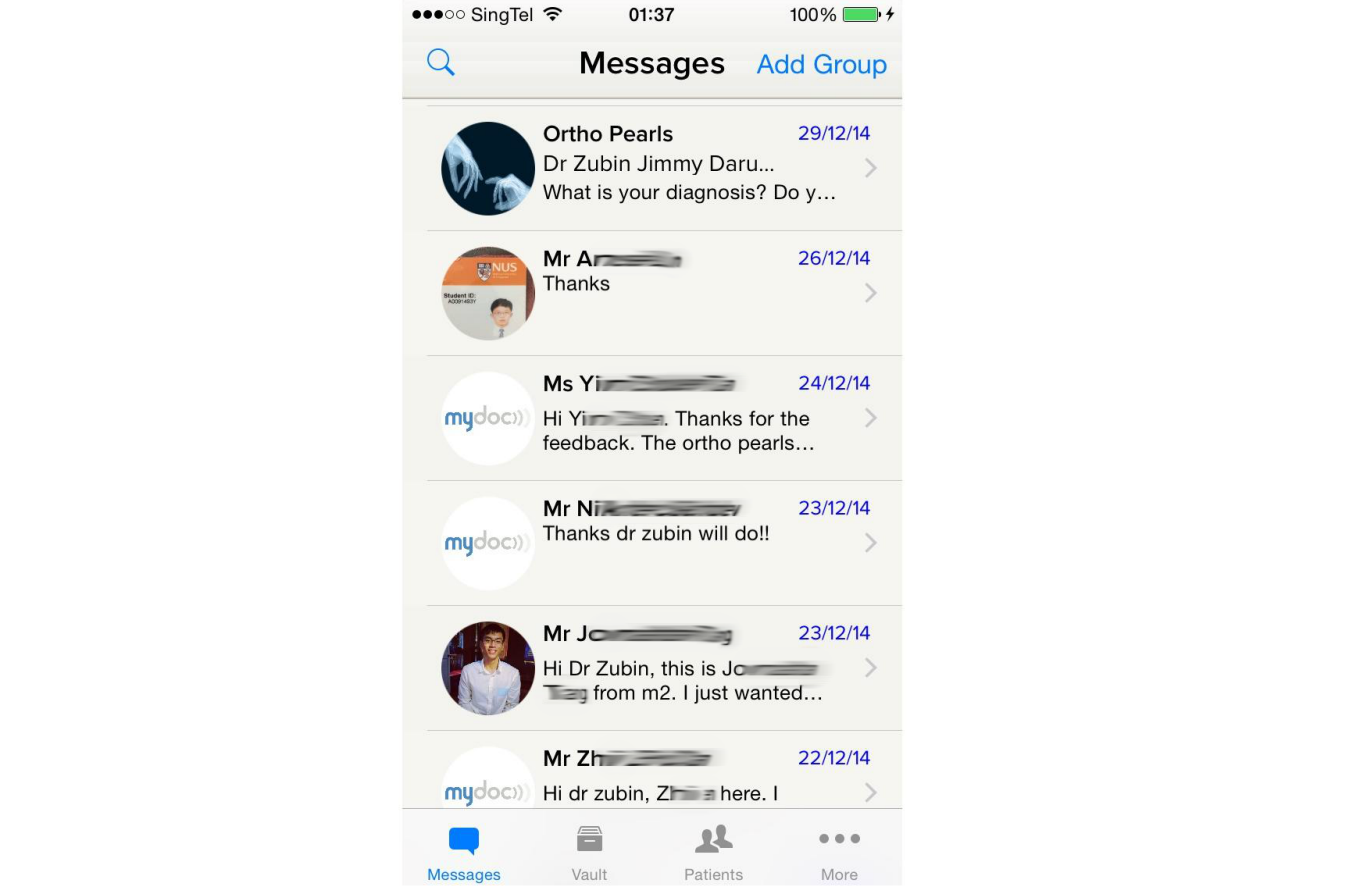
Secure communications interface showing personal messages.

**Figure 2:**
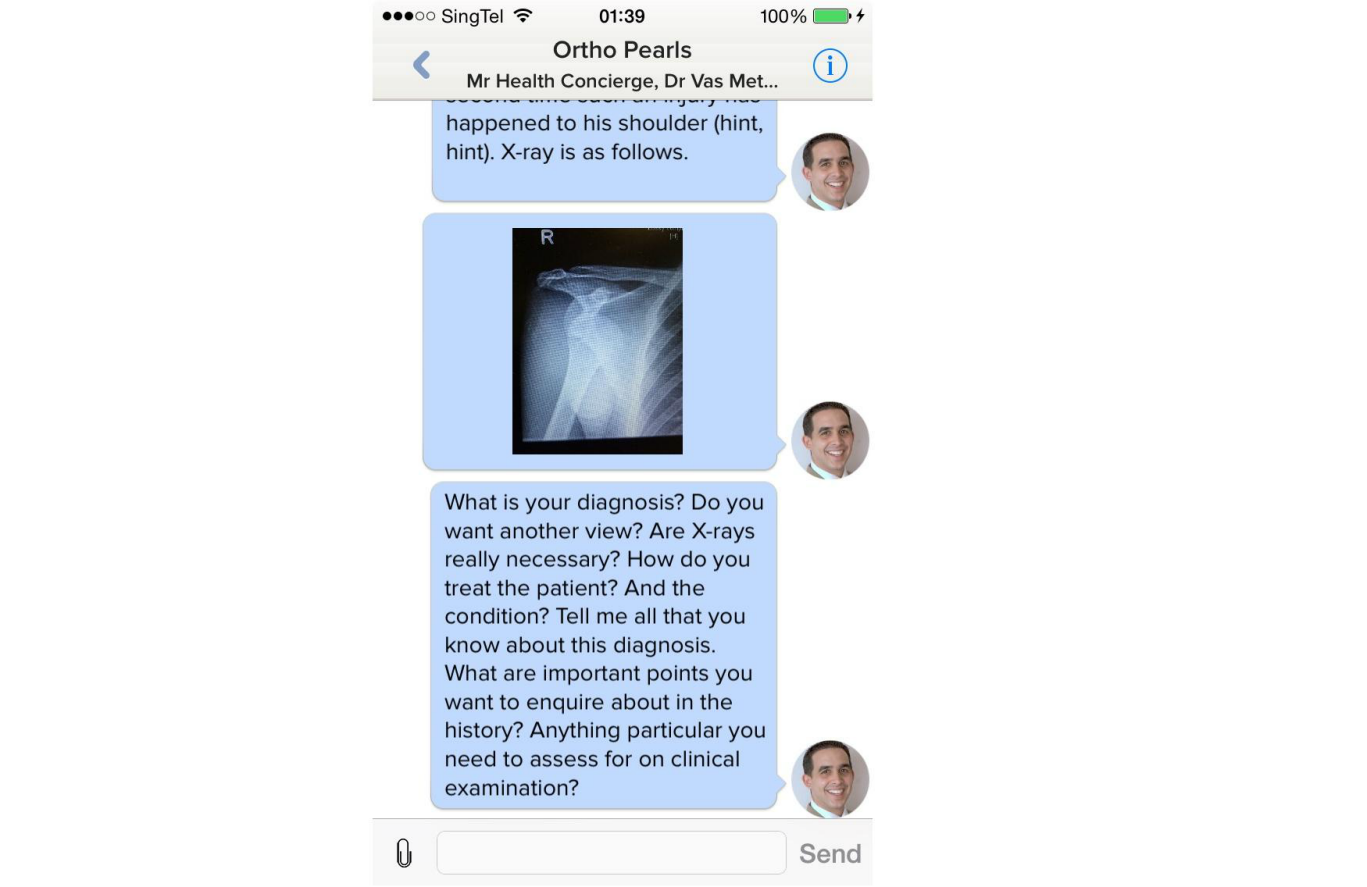
Screenshot depicting format of images used and questions asked as part of clinical case discussions.

**Figure 3:**
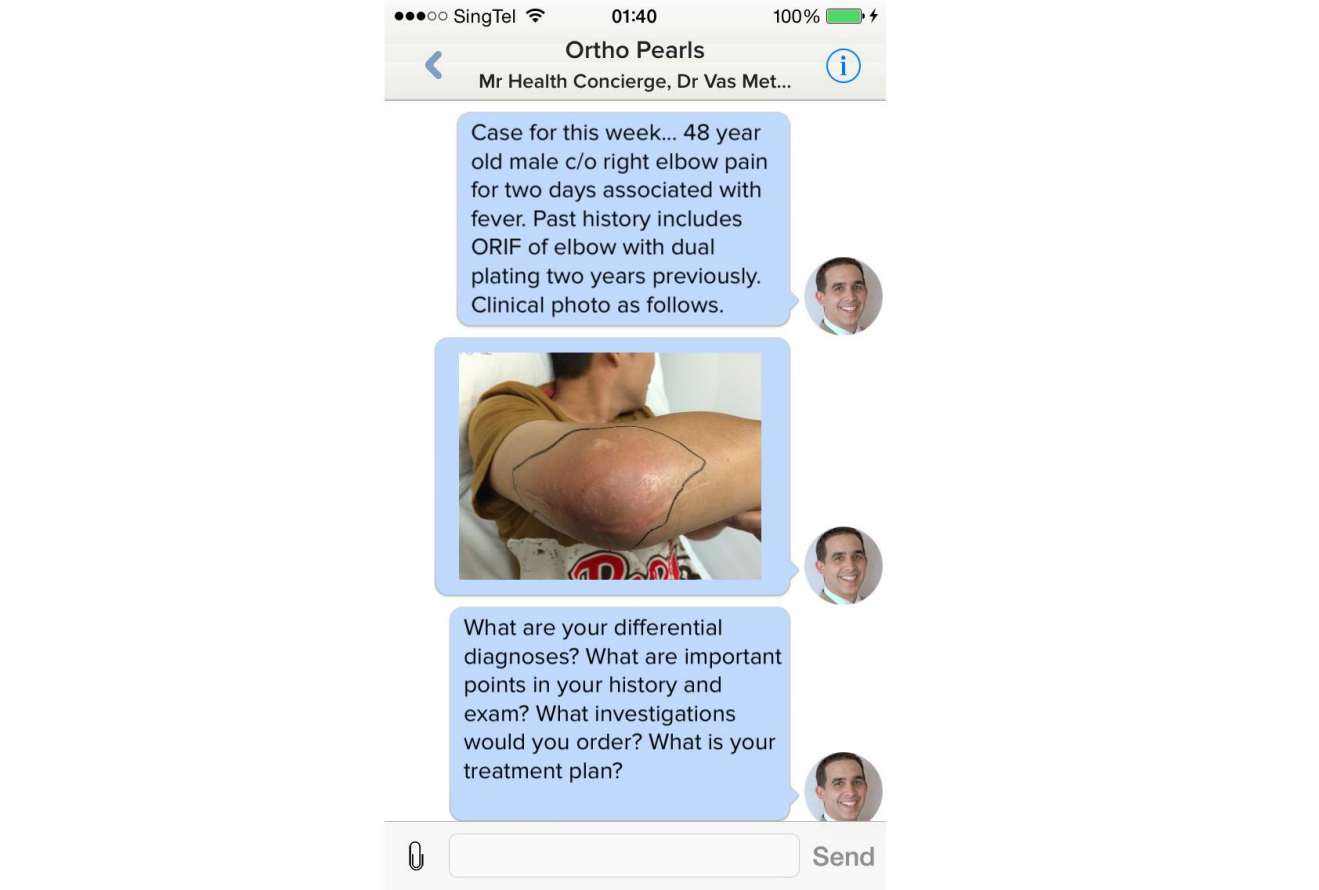
Sharing of a clinical photo of a patient with a common diagnosis all medical students rotating through orthopaedics are expected to know about as part of their syllabus.

Participants were asked to use MyDoc to replace or add to the current methods of communication through mobile applications, text messaging services, or social media. During the 6-week study period, students used MyDoc to communicate in the form of personal messages (Figure 1), case discussions (Figure 2), and providing patient details that peers might find interesting (Figure 3). At the end of 6 weeks, a constructed and validated questionnaire [4] was distributed to all study participants to gain feedback and determine student satisfaction with this innovative system.

## Results

All 240 students who participated in the study responded to the questionnaire. Two-thirds (159, 66.3%) considered MyDoc a secure communication platform among medical professionals; 15 (6.3%) disagreed and 66 (27.5%) stated they were unsure. The majority of students (154, 64.2%) felt that using MyDoc as a secure messaging platform is better than using current messaging systems and should be implemented school-wide in order to ensure the highest security standards and comply with the PDPA; 14 (5.8%) disagreed and 72 (30.0%) neither agreeing nor disagreeing. None of the students strongly disagreed. On being asked if they could easily communicate with their peers and senior doctors using the MyDoc secure messaging platform, 137 (57.1%) agreed or strongly agreed, 55 (22.9%) neither agreed nor disagreed, 40 (16.7%) disagreed, and 8 (3.3%) strongly disagreed. While the majority of students (140, 58.3%) were aware of and familiar with the PDPA of Singapore and its medicolegal implications prior to the study, all remaining students (100, 41.6%) attributed their subsequent awareness to this study. More than one-third of the students (91, 37.9%) did not feel MyDoc facilitated their learning in orthopedics while 149 (62.1%) said they felt it did or perhaps it did. In terms of the usefulness of clinical case discussions, the majority of students (122, 50.8%) found them to be useful or very useful while 24 (10.0%) did not. The remaining 94 students (39.2%) answered neutrally. The quality of the images was found to be poor by one student (0.004%), fair by 41 students (17.1%), good by 100 students (41.7%), very good by 63 students (26.3%), and excellent by 35 students (14.6%). In asking what role the clinical case discussions played in comparison to the students’ current modes of education, 208 (86.7%) felt it should complement current modes while 32 (13.3%) felt there was no role. Half (121) agreed or strongly agreed that MyDoc was user-friendly while 77 (32.1%) were unsure, 33 (13.8%) disagreed, and 9 (3.8%) strongly disagreed. On being asked if they felt using MyDoc to communicate with faculty would help build a stronger mentor-mentee relationship, 155 (64.6%) felt it would, 61 (25.4%) stated possibly, and 24 (10.0%) felt it would not. In addition, 96 students (40.0%) responded that they would recommend MyDoc to others, 37 (15.4%) said they would not, and 107 (44.6%) said they might. Over a 6-week period, 27,467 individual messages were sent between the students, equating to 654 per day. For group messages, a total of 19,751 were sent, equating to 470 per day.

Qualitative analysis showed 27 students (11.3%) considered *MyDoc* a secure platform due to it being only for doctors, stored on a secure data server, and described as a secure platform with emphasis on trustworthiness, confidentiality, and privacy. While 9 students (3.8%) questioned its reliability due to its nature of being a virtual platform on a mobile device, 6 (2.5%) considered it similar to the cross-platform smartphone messenger, WhatsApp. A total of 10 students (4.2%) were concerned with the technology glitches such as the intermittent system crashing, lack of message tracking, and intermittent malfunctioning on Android devices. From a spaced education point of view, the majority of students considered the telehealth platform secure, easy to use, and an educational tool that facilitated a deeper communication on clinical cases among peers and senior doctors. They commented that the clinical case discussions in the spaced format helped them apply knowledge to real clinical scenarios, provided extra sources of clinical information, built a platform for students to ask questions, and offered opportunities to learn from seniors and peers. The 41.6% (100) of students who were not aware of the PDPA all reported attributing their new-found awareness to this study. Among those who were already aware of the PDPA, they had learned about it through various channels, including talks given by the medical school or medical societies, parents or elders, newspapers, ethics classes, and research projects that required them to use secure platforms.

## Discussion

Our pilot study received positive feedback from most students with regard to using MyDoc’s secure application and platform. While the transmission of personal data in the healthcare sector is inevitable, not all methods in the marketplace are secure. This point is reiterated by the American Academy of Orthopaedic Surgeons, which clearly states that while texting speeds communication, it puts doctors at risk and increases liability because it is inherently nonsecure and noncompliant with safety and privacy regulations [5]. It should be noted that any service that simply sends text messages that are readily accessible to anyone who gains access to the phone the messages were sent to is not secure. Considering that the data sent within the medical community is often of a sensitive nature, it would be unacceptable to use any of these services. Although none of the authors of this manuscript condone the words or actions of the medical student who mocked a patient on Twitter some years ago [6], this example clearly illustrates the dangers of using a social media tool as a platform for transmitting any form of personal data.

The objectives of our pilot study were to assess the effectiveness of MyDoc as a secure communications tool and educational tool to further clinical case discussions and to raise awareness of the PDPA and its medicolegal implications to medical students by determining their reactions to this telehealth platform.

While the introduction and integration of MyDoc may prove to be advantageous in a number of ways, there are various disadvantages and key issues that need to be addressed. For example, the integrity of a secure wireless network used for the transmission of sensitive and confidential information is paramount. From a practical point of view, possible loss of the Internet connection or network may make the use of the system both impractical and frustrating. Lastly, the speed of message transmission was an issue a number of study participants raised, stating that it is not as fast as other applications. However, it must be understood that unlike other systems in the marketplace where messages can be read by anyone, forwarded to anyone, remain unencrypted on telecommunication provider servers, and most importantly stay forever on the phones of senders and receivers [5], messages on MyDoc are not stored on the phone. That together with the various levels of security account for the slightly slower transmission speed.

There are many potential uses of MyDoc in medical schools, including the following:

- Enhance and complement current student interactive sessions
- Mentorship for student houses and groups
- Specialty-specific groups for clinical years
- Clinical case-based discussions for clinical as well as preclinical years, (including a focus on basic sciences to allow constant refreshing of memory as students progress from the preclinical to clinical years)
- Access to faculty or teaching staff to answer subject and/or specialty-based questions, including in real time during lectures with the option of anonymity
- Provision of and sharing patient details of interesting patients with clinical signs
- Secure messaging and compliance with the PDPA and medical privacy laws of other countries
- Dissemination of class or faculty announcements
- Observation of outpatient clinics through the use of telemedicine in the form of live video feeds

While some may argue and raise the question as to whether the sharing of identifiable data is even necessary in the context of medical education, we agree but only to a limited extent. Often what one may consider nonidentifiable (a tattoo, for example) may in fact and on the contrary be very identifiable should there be a particular uniqueness to it. In the case of taking a photo of an x-ray or lab result, a name or serial number may invariably but unknowingly be captured in the corner of the image. Ensuring this data is transferred via a secure and PDPA-compliant platform is advantageous to all parties concerned. And for these same reasons, the need for compliance with privacy laws becomes clear.

A few years ago, an article stated that telehealth is expected to grow 6-fold by 2017 [7]. Subsequently, it has been explained how it may even act as a remedy for chronic hospital readmissions [8] and why virtual doctor visits are better than in-person ones [9]. With our pilot study having excellent results in terms of acceptance and satisfaction from all medical students with regard to using MyDoc, the integration of the application in a medical school setting to provide a variety of functions has exciting and limitless potential and is something all medical schools should consider, because our students of today are our doctors of tomorrow. More so with the recently introduced PDPA, this secure application and platform’s versatility in terms of its potential applications is something medical schools may find worth promoting, the latter clearly evident by the lead author’s recent success in being awarded a Learning Innovation Fund–Technology (LIFT) grant by the National University of Singapore to use technology in medical education to seek feedback from medical students.

## Abbreviations

PDPA: Personal Data Protection Act
